# Modulating the Growth, Antioxidant Activity, and Immunoexpression of Proinflammatory Cytokines and Apoptotic Proteins in Broiler Chickens by Adding Dietary *Spirulina platensis* Phycocyanin

**DOI:** 10.3390/antiox11050991

**Published:** 2022-05-19

**Authors:** Anaam E. Omar, Hanan S. Al-Khalaifah, Ali Osman, Ahmed Gouda, Shimaa I. Shalaby, Elshimaa M. Roushdy, Samar A. Abdo, Sozan A. Ali, Aziza M. Hassan, Shimaa A. Amer

**Affiliations:** 1 Department of Nutrition and Clinical Nutrition, Faculty of Veterinary Medicine, Zagazig University, Zagazig 44511, Egypt; eaibrahym@vet.zu.edu.eg; 2 Environment and Life Sciences Research Center, Kuwait Institute for Scientific Research, P.O. Box 24885, Safat, Kuwait City 13109, Kuwait; hkhalifa@kisr.edu.kw; 3 Biochemistry Department, Faculty of Agriculture, Zagazig University, Zagazig 44511, Egypt; aokhalil@zu.edu.eg; 4 Animal Production Department, Agricultural & Biological Research Division, National Research Center, Dokki, Cairo 11865, Egypt; ag.abdullah@nrc.sci.eg; 5 Physiology Department, Veterinary Medicine Faculty, University of Zagazig, Zagazig 44511, Egypt; siabdallah@zu.edu.eg; 6 Animal Wealth Development Department, Faculty of Veterinary Medicine, Zagazig University, Zagazig 44511, Egypt; emroshdy@zu.edu.eg; 7 Biochemistry Department, Faculty of Veterinary Medicine, University of Zagazig, Zagazig 44511, Egypt; saaabdallah@vet.zu.edu.eg; 8 Department of Histology and Cytology, Faculty of Veterinary Medicine, Zagazig University, Zagazig 44511, Egypt; sozanismaeil@zu.edu.eg; 9 Department of Biotechnology, College of Science, Taif University, P.O. Box 11099, Taif 21944, Saudi Arabia; a.hasn@tu.edu.sa

**Keywords:** broiler chickens, growth performance, phycocyanin, proinflammatory cytokines, gut histology

## Abstract

This study investigated the dietary effect of *Spirulina platensis* phycocyanin (SPC) on growth performance (body weight (BW), body weight gain (BWG), feed intake (FI), feed conversion ratio (FCR)) at starter, grower, and finisher stages, intestinal histomorphology, serum biochemical parameters, inflammatory and antioxidant indices, and proinflammatory cytokines (tumor necrosis factor-α and caspase-3) immune expression in broiler chickens. In total, 250 one-day-old chicks (Ross 308 broiler) were randomly allotted to five experimental groups (5 replicates/group, 10 chicks/replicate) and fed basal diets supplemented with five levels of SPC (0, 0.25, 0.5, 0.75, and 1 g kg^–^^1^ diet) for 35 days. Compared with SPC0 treatment, different SPC levels increased the overall BW and BWG without affecting the total feed consumption. However, the FCR decreased linearly with an increase in supplementation level. The serum levels of total proteins, albumin, globulins, and growth hormone increased linearly by increasing levels of SPC supplementation. Further, SPC supplementation increased the thyroxin hormones without affecting serum glucose and leptin levels. Serum total cholesterol (TC) and low-density lipoprotein cholesterol (LDL-C) values decreased in broilers fed SPC0.250 and SPC1 diets. Triglycerides (TG) decreased in SPC0.25-, SPC0.75-, and SPC1-treated groups. Though antioxidant enzyme activities (total antioxidant capacity, catalase, and superoxide dismutase) increased linearly and quadratically, malondialdehyde (MDA) decreased linearly by increasing the SPC level. There was no effect on serum proinflammatory cytokines IL1β levels. Immunolabelling index of caspase-3 and tumor necrosis factor-α (TNF-α) were downregulated by SPC supplementation. The intestinal histomorphology is represented by increased villus height, the villus height to crypt depth ratio, and numbers of goblet cells in different sections of the small intestine. In conclusion, SPC supplementation is beneficial in broiler chicken diets due to its growth-promoting, antioxidant, and anti-inflammatory properties.

## 1. Introduction

There is a growing need for alternative strategies to replace synthetic components with natural antimicrobial compounds in poultry diets to improve growth performance, enhance immunity, reduce oxidative stress, and improve gut histology and digestibility. The extensive use of synthetic antimicrobials in poultry diets causes drug-resistant microbial growth, impacting human and bird health [1,2,3].

Microalgae are photosynthetic prokaryotic or eukaryotic organisms that convert sunlight, water, and CO_2_ to algal biomass. Some species are rich in carbohydrates, minerals, proteins, and other essential compounds. The nutrient value is influenced by algae kind, culture media composition, incubation time and temperature, and environmental conditions [4].

*Spirulina* is a blue-green filamentous photosynthetic alga, containing 55–70% proteins, 15–25% carbohydrates, 18% essential fatty acids, minerals, vitamins, carotenes, chlorophyll-a, and phycobiliprotein pigments (phycocyanin, phycoerythrin, and allophycocyanin). Moreover, it is rich in phenolic acids and gamma-linoleic acid [5]. *Spirulina platensis* and *Spirulina maxima* are the most important *Spirulina* species and are cultured commercially in several countries [6]. *S. platensis* has several health benefits, including immunity stimulation and growth parameters with hypolipidemic, anti-inflammatory, and antioxidative activities [7,8].

Phycocyanin (PC) is a blue photosynthetic pigment in cyanobacteria and some red algae of the phycobiliprotein family. It is water-soluble, located in the cytoplasmic membrane, and released outside when the thylakoid membrane is destroyed by lysozyme enzyme and EDTA chelate cations [9,10]. PC can be extracted using organic and inorganic solvents, ultrasound, enzyme homogenization, freezing, and thawing from *Spirulina* [11,12]. PC possesses antioxidant, radical scavenging, anti-inflammatory, antiarthritic, hepatoprotective, antitumor, and immune-enhancing properties [6,13]. The antioxidant activity of PC is attributed to alkyl, hydroxyl, and peroxyl radical scavenging activity due to their hydroxyl and aromatic substituent structures. For their health-stimulating characteristics, natural antioxidants are preferred in broilers production [14] as they reduce reactive oxygen species (ROS) production and subsequent oxidative stress [15]. PC is a safe, natural antimicrobial product [16,17].

To the best of our knowledge, studies on SPC in poultry are limited. Hence, this study aimed to evaluate the effects of dietary supplementation of different SPC levels on growth, intestinal histomorphology (an indicator of gut health), blood biochemical parameters, antioxidant status, and immunoexpression of proinflammatory cytokines and apoptotic proteins in broiler chicken.

## 2. Material and Methods

### 2.1. Isolation and Purification of Spirulina Platensis Phycocyanin

#### 2.1.1. Micro-Organism Source

*S. platensis* was acquired from the Microbiology Department, Faculty of Agriculture, Zagazig University, Zagazig, Egypt.

#### 2.1.2. Culture Reparation

The *S. platensis* cultures were grown in a nitrogen-free medium (BG^0^11). The fresh *S. platensis* culture was prepared by inoculating 250 mL of BG^0^11 medium with 10 mL of 10 days old culture in 500 mL Erlenmeyer flasks. Inoculated flasks were incubated at 26 ± 2 °C for 28 days under continuous illumination (600–800 lux) using a 36 W white fluorescent lamp.

#### 2.1.3. Phycocyanin Extraction and Purification

PC was extracted from *S. platensis* fresh biomass according to Sarada et al. [18] and Salama et al. [19]. *S. platensis* cells (after 30 days of growth) were harvested by centrifugation at 3000× *g* for 5 min (Jouan, MR 18-22, Saint-Herblain, France) at 20 °C. Separated cell pellets were washed in 1M Tris–HCl buffer (pH 8.1) (Sigma-Aldrich, Darmstadt, Germany). One volume of washed cell mass was re-suspended in five volumes of the same buffer and treated for the extraction of PC using the freeze–thaw method (freezing at –50 °C and thawing at 25 °C). The resulting suspension was centrifuged for 10 min at 5000× *g* to separate the supernatant (the crude extract) and kept in the refrigerator. The pigment absorption was measured using UV/VIS Spectrophotometer (JENWAY, Cole-Parmer, Staffordshire, England) at 620, 640, and 650 nm against 0.05 M phosphate buffer as blank. To obtain 20% saturation, powdered ammonium sulfate was gradually added to the crude extract of PC and kept under continuous stirring for 1 h. The resulting solution was stored overnight and centrifuged at 17,000× *g* for 20 min. As previously described, the resulting supernatant was pooled and subjected to 70% ammonium sulfate saturation. After overnight incubation, the solution was centrifuged at 17,000× *g* for 20 min, and the resulting pellets were re-suspended in a small quantity of 20 mM Tris–HCl buffer (pH 8.1) and subjected to dialysis for 48 h against the same buffer, with changes of buffer four times. Next, it was lyophilized and stored at –20 °C for further use.

To determine the absorbance of the sample and PC estimation [20], the following equations were used:

C-PC (mg/mL)=(OD620−0.7OD650)/7.38;

C-APC (mg/mL)=(OD650−0.19OD620)/5.65;

C-PE(mg/mL)=(OD640−2.8[C-PC]−1.34[C-APC])/12.7.

### 2.2. Birds

The experimental trial was conducted in the Poultry Research Unit of the Faculty of Veterinary Medicine, Zagazig University, Egypt. The experiment procedures were approved by the Institutional Animal Care and Use Committee (ZU-IACUC) of Zagazig University, Egypt (Approval No. ZU-IACUC/2/F/16/2022). A total of 250 one-day-old Ross 308 broiler chicks were procured from a local hatchery. They were incubated on neomycin broad-spectrum antibiotic and dehydrated solution for three days, reaching an average initial weight of 89.46 ± 0.85 g. The chicks were raised in an open, ventilated house with suitable litter. During the first week, the room temperature was controlled at 34 °C and was gradually decreased until reaching 25 °C at the end of the rearing stage. Standard health and vaccination programs were conducted against Newcastle and Gumboro diseases. Chicks were observed daily, and notes were taken for any health issues.

### 2.3. Experimental Design and Diets

The chicks were randomly allotted into five experimental groups (50 chicks each, 5 replicates/group, 10 chicks/replicate). The experimental treatments were as follows: T1, control group (basal diet without SPC addition (SPC0); T2, basal diet + 0.25 g SPC kg^–^^1^ diet (SPC0.25); T3, basal diet + 0.5 g SPC kg^–^^1^ diet (SPC0.5); T4, basal diet + 0.75 g SPC kg^–^^1^ diet (SPC0.75); T5, basal diet + 1 g SPC kg^–^^1^ diet (SPC1). Feed and water were added *ad libitum* for 35 days of trial. As shown in Table 1, rations were provided in the mashed form and were formulated according to Ross’s manual guide, AVIAGEN [21].

### 2.4. Growth Performance

Birds were weighed individually on the 4th day of age to determine the average initial BW and then weighed at 10, 23, and 35 days to calculate the average BW of the birds in each group. At each interval, the BWG was determined as the difference between the final and the initial BW. The feed intake (FI) and feed conversion ratio (FCR) were calculated as per the following equations:

$$\mathrm{Feed}\;\mathrm{intake}\;\left(\mathrm{FI}/\mathrm{bird}\right)=\frac{\mathrm{the}\;\mathrm{amount}\;\mathrm{of}\;\mathrm{feed}\;\mathrm{offered}\;-\;\mathrm{feed}\;\mathrm{residues}}{\mathrm{No}.\;\mathrm{of}\;\mathrm{birds}\;\mathrm{in}\;\mathrm{each}\;\mathrm{replicate}}$$

$$\mathrm{Feed}\;\mathrm{conversion}\;\mathrm{ratio}\;\left(\mathrm{FCR}\right)=\frac{\mathrm{feed}\;\mathrm{intake}\;\left(g\right)}{\mathrm{weight}\;\mathrm{gain}\;\left(g\right)}.$$

### 2.5. Sampling

Three birds were randomly selected from each replicate (*n* = 15/group) and euthanized by cervical dislocation, according to the American Veterinary Medical Association guidelines [22]. Then, blood samples were collected into sterilized tubes without anticoagulant and were at room temperature for clotting. The samples were then centrifuged (at 3500 rpm for 15 min) to separate the serum. The serum samples were kept at −20 °C in Eppendorf tubes until chemical analysis. For histological and immunohistochemical examination, the small intestine and liver samples were collected.

### 2.6. Blood Biochemical Indices

The total protein serum level was determined according to Grant [23], and the serum albumin level was evaluated as per Doumas et al. [24]. The serum globulin level was calculated by subtracting albumin values from total proteins [25]. The growth hormone (GH) was determined according to the instructions provided in the chicken ELISA kit of MyBioSource Co., San Diego, CA, USA with Cat. No. MBS266317. 

According to the procedures of McGowan et al. [26] and Allain et al. [27], the serum triglycerides (TG) and total cholesterol (TC) were determined, respectively, with diagnostic kits of Spectrum BioScience (Egyptian Company for Biotechnology, Cairo, Egypt). Serum high-density lipoprotein cholesterol level (HDL-C) was measured using Vassault et al. [28] methodology. LDL-C was estimated using the following Iranian formula:

LDL-C=TC/1.19+TG/1.9 − HDL/1.1−38.

### 2.7. Inflammatory and Antioxidant Indices

To quantify interleukin 1β (IL1β) (Cat. No. MBS2024496), specific ELISA assay kits (MyBioSource, San Diego, CA, USA) were used. According to the methodology of McDonald and Hultin [29], Rice-Evans and Miller [30], Aebi [31], and Nishikimi et al. [32], Malondialdehyde (MDA), the total antioxidant capacity (TAC), catalase (CAT) activity, and superoxide dismutase (SOD) were estimated, respectively, using MyBioSource ELISA Kits (Cat. Nos. MBS2700234, MBS038818, and MBS705758, respectively). 

### 2.8. Histological and Immunohistochemical Examination

For analysis, two-centimeter samples (three/group) were taken from the small intestine (duodenum, jejunum, and ileum) and were fixated in neutral buffered formalin (10%). Briefly, specimens were subjected to ascending grades of ethanol (75–100%) for dehydration. These specimens were placed in xylol I and II, embedded in paraffin, sliced with a microtome (Leica RM 2155, Wetzlar, Germany) into 4 µm cross-sections and longitudinal sections, and stained using hematoxylin and eosin (H&E) [33]. Images of each animal in each group (25 images for each group) were captured with an AmScope 5.0 MP microscope digital camera (AmScope, Irvine, CA, USA) in a low-power field (40× magnification). Intestinal villus height (VH) was measured (µm) tip to the base of the villus, and the crypt depth (CD) was calculated from the villus—crypt junction to the distal limit of the crypt. The VH, villus width (VW), CD, and goblet cell count (GCC) per area of epithelium layer were measured using Mitocam^®^ software (Motic Images plus 2.0, Hong Kong, China).

According to Saber Saber et al. [34], liver samples (three samples/group) were collected to examine the immunoexpression of caspase-3 and TNF-α at the end of the experiment. To incubate the tissues, an endogenous peroxidase blocking reagent containing hydrogen peroxide and sodium azide (DAKO peroxidase blocking reagent, Cat. No. S 2001) was used. Then, 1–2 drops of the supersensitive primary monoclonal antibody against caspase-3 and TNF-α (Cat. Nos. NB100–56708 and NB600–587, respectively, Novus Biologicals, Briarwood Avenue, USA) were added to these sections. The slides were then counterstained using hematoxylin and visualized under the microscope. ImageJ software (ImageJ bundled with 64-bit Java 1.8.0_172) was used to estimate the percentages of immunostaining positive area at high magnification (100×) in five sections per group, according to Rizzardi et al. [35].

### 2.9. Statistical Analysis

ANOVA was applied based on polynomial orthogonal contrasts. SPSS Version 17 for Windows (SPSS Inc., Chicago, IL, USA) calculated the linear and quadratic regression equations. Duncan’s multiple range test verified the significant difference between mean values, and the significance level was set at *p* < 0.05. 

## 3. Results

### 3.1. Phycocyanin Characterization

**Figure 1:**
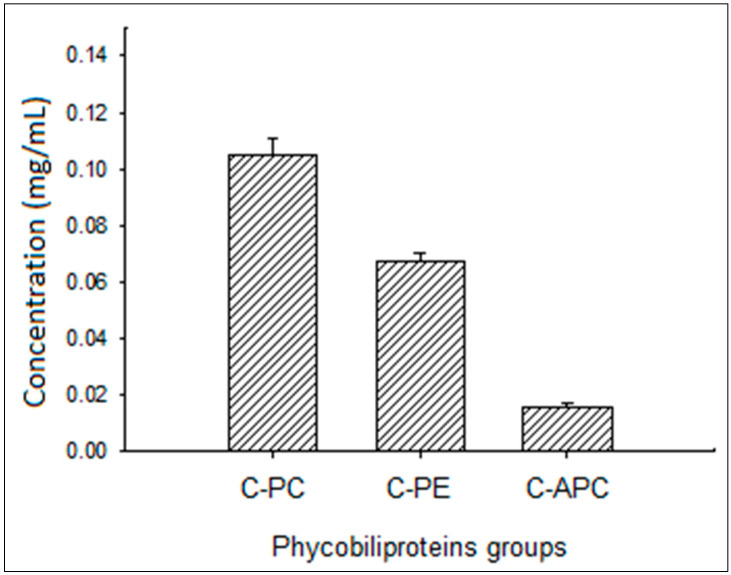
Cyanobacterial phycobiliproteins group concentration (mg/mL): phycocyanin (C-PC), phycoerythrin (C-PE), and allophycocyanin (C-APC).

The three PC components (Figure 1) with the highest concentrations were located in the following order: PC (0.105 mg/mL) > phycoerythrin (0.067 mg/mL) > allophycocyanin (0.016 mg/mL).

### 3.2. Growth Performance 

Table 2 presents the growth parameter data. Body weight (BW) and body weight gain (BWG) was linearly increased (*p* < 0.01) throughout the starter period in broilers fed 1 g/kg SPC-supplemented diets. Different SPC levels did not affect the FCR and FI compared to the SPC0 treatment (*p* > 0.05). BW and BWG increased linearly in chickens given SPC0.75 treatment (*p* ≤ 0.01) during the grower period. However, those fed SPC0.25, SPC0.5, and SPC1 diets showed nonsignificant improvement compared to SPC0 treatment. FCR linearly decreased in chicken given SPC0.5, SPC0.75, and SPC1 treatments and linearly increased under SPC0.25 treatment (*p* < 0.01). Moreover, all SPC levels did not affect the average FI (*p* = 0.10) compared with the SPC0 treatment. Different SPC levels linearly and quadratically increased (*p* < 0.01) BW and BWG with no effect on the total FI during the final and overall periods. Overall, FCR linearly decreased (*p* = 0.02) in SPC0.5, SPC0.75, and SPC1 groups. Further, final BW was the highest among broilers fed diets supplemented with SPC at 0.75 g/kg and the least in the SPC0 group.

### 3.3. Serum Biochemical Indices

The effect of SPC addition on the serum biochemical parameters of broilers is highlighted in Table 3. Total proteins (*p* < 0.01), albumin (*p* < 0.01), total globulins (*p =* 0.02), and GH levels were linearly increased by different SPC level supplementations (*p* < 0.01). These changes were greater in broilers fed a 1 g/kg SPC-supplemented diet than those fed a 0.75 g/kg SPC diet. Meanwhile, serum glucose and leptin levels were not significantly changed (*p* > 0.05). Thyroxin hormones (T3 and T4) were increased linearly and quadratically in all SPC treatment groups compared with the SPC0 treatment group (*p* < 0.01).

### 3.4. Lipid Profile

Serum TC and LDL values decreased linearly in broilers fed ration supplemented with SPC at 0.25 or 1 g/kg diet. In contrast, serum triglyceride decreased linearly in groups given SPC0.250, SPC0.750, and SPC1 treatments (*p* < 0.05). All supplementation levels had no significant effect on high-density lipoprotein cholesterol (HDL-C) and very low-density lipoprotein cholesterol (VLDL-C) (*p* > 0.05) when compared with the control group (Table 4).

### 3.5. Inflammatory and Antioxidant Indices

The impact of SPC on the activities of serum antioxidant enzymes of broiler chickens is shown in Table 5. TAC, catalase (CAT), and superoxide dismutase (SOD) activities increased linearly and quadratically in all SPC treatment groups (*p* < 0.05) in comparison with the SPC0 treatment group. The higher supplementation levels observed the highest results. Moreover, serum MDA linearly decreased in groups fed diets supplemented with SPC at 0.5, 0.75, and 1 g/kg diets. SPC addition did not affect IL1β levels.

### 3.6. Histological Findings and Morphometric Measures

**Figure 2:**
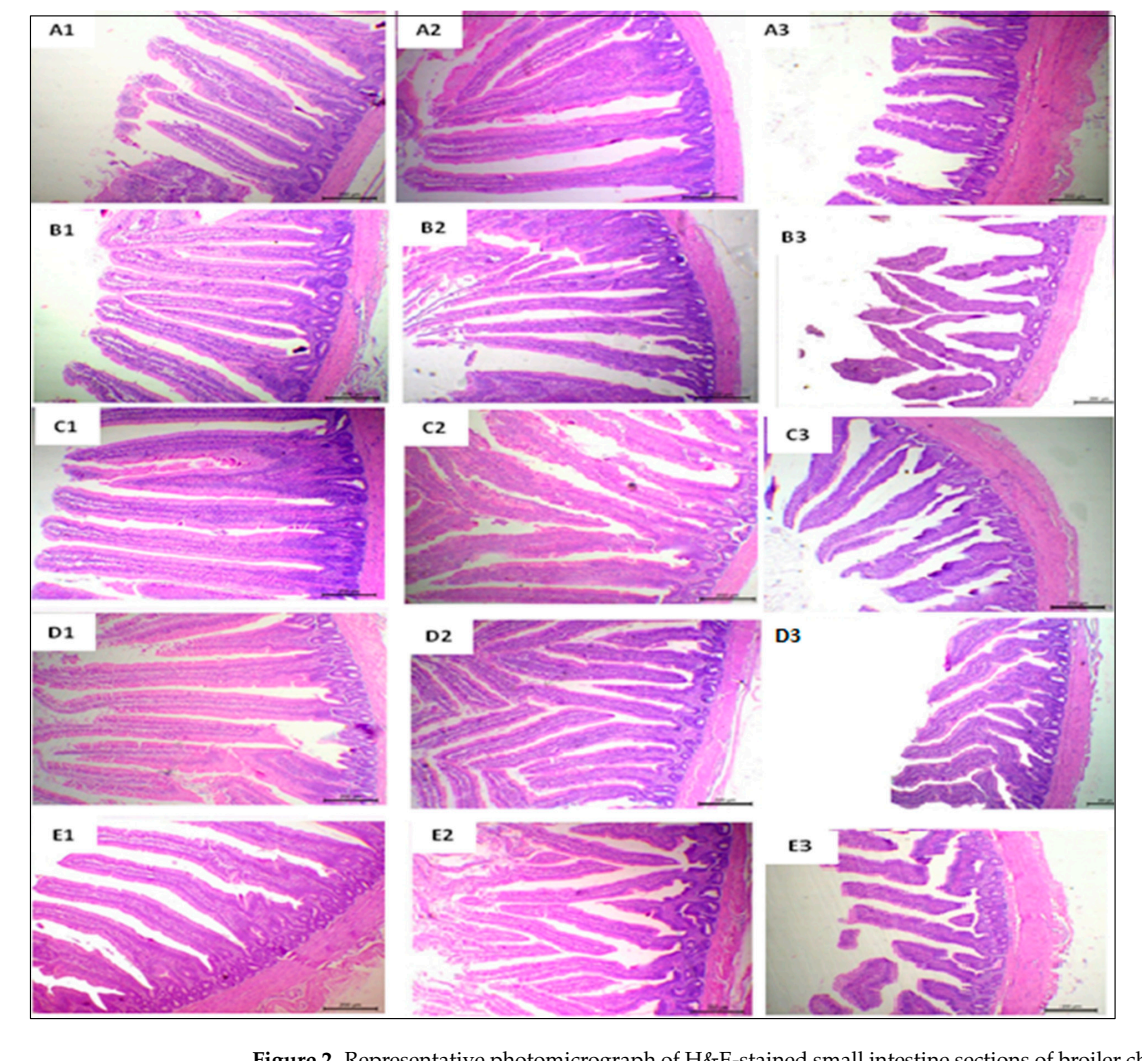
Representative photomicrograph of H&E-stained small intestine sections of broiler chickens in 40× magnification. Duodenal sections from SPC0 showed normal intestinal villi with a free lumen (**A1**); those from SPC0.25 group showed a mild increase in villus height (**B1**); sections from SPC0.5–1 groups revealed markedly thin, tall, and separate villi with mild goblet cell proliferation, increased sizes, and rows of enterocytes with arranged lamina propria (**C1**,**D1**,**E1**). Jejunal segment sections of SPC0 showed free lumina with nearly normal villus structures (**A2**), while sections from the SPC0.25 group showed a small increase in villi length (**B2**). Jejunum segments from SPC0.5−1 groups showed closely packed villi with a marked increase in their length and markedly serrated surfaces with goblet cell metaplasia (**C2**,**D2**,**E2**). Ileal segment sections from basal treatment showed tongue-shaped villi with a different height (**A3**); sections from the SPC0.25 group exhibited normal histology (**B3**); the sections from the SPC0.5 and SPC0.075 groups showed relatively increased villus height (**C3**,**D3**); those from the SPC1 diet group showed normal villi (**E3**).

Figure 2 presents a representative photomicrograph of small intestine sections stained with H&E of broilers with 40× magnification. Normal villi with a free lumen were observed in the slices of the duodenal segments from the SPC0 group. A mild increase in villus height was shown in the SPC0.25 group. In contrast, the SPC0.5–1 groups showed prominently tall, thin, and distinct villi with a mild proliferation of goblet cells, along with an enlarged size, and rows of enterocytes with arranged lamina propria. Free lumina with normal villous structures were exhibited in segments from the jejunal sections of the SPC0 group, whereas sections from the SPC0.25 group showed a small increase in villi height. SPC0.5, SPC0.75, and SPC1 jejunum segments showed closely packed villi with a significant increase in length and distinctly serrated surfaces with goblet cell metaplasia. Ileal segment samples from the basal control treatment showed tongue-shaped villi with different heights. The SPC0.25 group, on the other hand, showed normal histology, while the SPC0.5 and SPC0.75 groups had significantly increased villus height, and the SPC1 group showed normal villi.

Table 6 shows the results of intestinal morphometric measurements. In comparison to the SPC0 treatment group, there was a linear and quadratic increase in the villus height (VH), crypt depth (CD), VH: CD ratio, and numbers of goblet cells in different sectors of the small intestine observed in all SPC treatment groups (*p* < 0.01). Different SPC supplementation levels had no effect on the duodenal and jejunal villus width. However, ileal villus width increased linearly in the SPC0.25 and SPC1 treatment groups (*p* < 0.01). 

### 3.7. Immunohistochemical Analysis and Morphometric Measures

**Figure 3:**
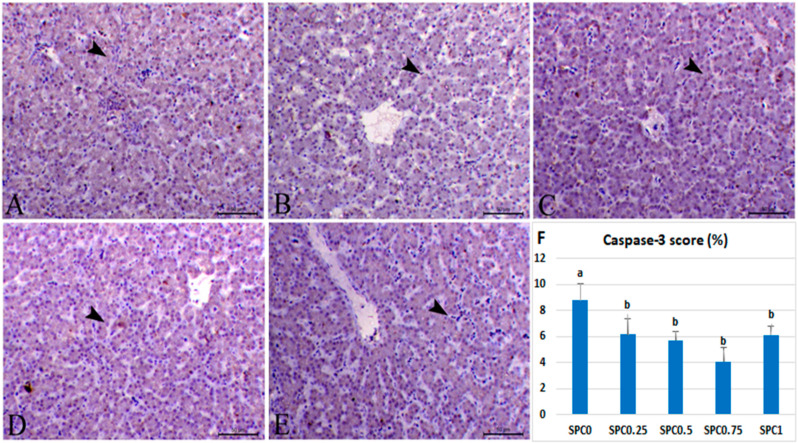
Photomicrograph of hepatic tissues immunostained with caspase-3 antibody. Liver of control basal group showing mild cytoplasmic expression of caspase-3 within hepatocytes (**A**) (arrowhead). Liver of SPC0.25 group showing mild expression of caspase-3 within hepatocytes (**B**) (arrowhead). The liver of the SPC0.5 group showed a decrease in the cytoplasmic immunostaining of caspase-3 within hepatocytes (**C**) (arrowhead). Liver of SPC0.75 group showing mild cytoplasmic expression of caspase-3 within hepatocytes (**D**) (arrowhead). Liver of SPC1 group showing mild cytoplasmic expression of caspase-3 within hepatocytes (**E**) (arrowhead). Bar = 50 µm. (**F**) shows morphometric measures of caspase-3 immunostaining expression (%). ^a,b^ Means carrying different superscripts were significantly different (*p* ≤ 0.05).

**Figure 4:**
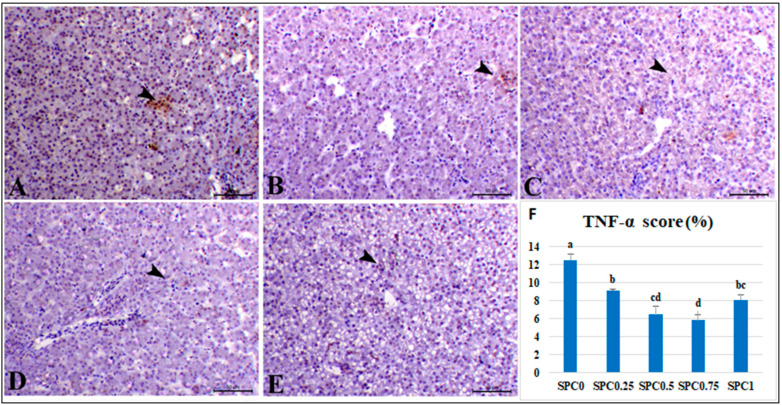
Photomicrograph of hepatic tissues immunostained with TNF-α antibody. The liver of the control SPC0 group showed mild expression of TNF-α within a few hepatocytes (**A**) (arrowhead). The liver of the SPC0.25 group showed scanty expression of TNF-α within hepatocytes (**B**) (arrowheads). The liver of the SPC0.5 group showed a marked decrease in immunostaining of TNF-α within hepatic tissues (**C**) (arrowhead). The liver of the SPC0.75 showed a significant decrease in the expression of TNF-α within the blood sinusoids (**D**) (arrowhead). The liver of SPC1 showed a marked decrease in the expression of TNF-α within hepatocytes (**E**) (arrowhead). Bar = 50 µm. (**F**) shows morphometric measures of TNF-α immunostaining expression (%). ^a,b,c,d^ Means carrying different superscripts were significantly different (*p* ≤ 0.05).

Figure 3F and Figure 4F demonstrate the morphometric scores of the immune staining expression of caspase-3 and TNF-α antibodies in the liver. Immunolabelling index percentage of caspase-3 and TNF-α were lowered linearly and quadratically in all SPC-supplemented groups (*p* < 0.05), with the lowest value in the SPC0.75 group, followed by the SPC0.5 group.

Figure 3 shows the photomicrograph of hepatic tissues immunostained with caspase-3 antibody. The liver of the control group revealed the mild cytoplasmic appearance of caspase-3 within the hepatic cells (8.77%). The SPC0.25 group showed mild caspase-3 expressions within hepatocytes (6.17%), and the SPC0.5 group demonstrated a decrease in the cytoplasmic immunostaining of caspase-3 within hepatocytes (5.7%). The SPC0.75 and SPC1 groups exhibited mild cytoplasmic manifestation of caspase-3 within hepatocytes (4.08 and 6.13%, respectively).

Figure 4 shows a photomicrograph of hepatic tissues immunostained with TNF-antibodies. TNF-expression was mild within a few hepatocytes (12.5%) in the liver of the SPC0 group, but TNF-expression was scanty within hepatocytes in the liver of the SPC0.25 group (9.08%). SPC0.5 and SPC1 groups showed a significant decrease in immunostaining of TNF-α within hepatic tissues (6.47 and 8.08%). SPC0.75 group showed a marked reduction in TNF-α expression within the blood sinusoids (5.83%).

## 4. Discussion

### 4.1. Growth Performance

The addition of dietary *S. platensis* PC positively affects the final BW and BWG of broilers chickens without influencing FI and FCR. The SPC0.75 treatment group showed the best outcomes, followed by the SPC0.5 group. This advance in growth parameters may be due to the improvement in birds’ health, evidenced by the small intestine morphology with greater villi length, increasing numbers of goblet cells, and enhancement in their absorption surface, resulting in enhanced nutrient digestibility and absorption. SPC has been shown to increase antioxidant enzyme activity while lowering proinflammatory cytokines (IL1β and IFN-γ) production. It has a positive effect on gastrointestinal flora and increases the activities of digestive enzymes, resulting in enhanced total tract digestibility of dry matter and nitrogen [36]. Furthermore, it improves nutrient digestibility of amino acids and protein synthesis [37] and apparent metabolizable energy digestibility [38], as well as positively modifying the intestinal microbial population by decreasing pathogenic bacteria such as *E. coli* and increasing lactic acid bacteria [39,40]. PC stimulates short-chain fatty acid production and reduces intestine pathogens, resulting in gut health improvement [41]. High essential amino acid contents of *Spirulina* are critical for improving health status and BW, as well as decreasing health disorders and effects of heat stress [42].

Furthermore, PC is a hydrophilic protein that regulates vascular colloidal osmotic pressure to maintain equilibrium with bodily fluids [43,44]. PC possess antioxidant, anti-inflammatory, and immune-boosting properties [41,42,45]. *Spirulina* extracts have antimicrobial effects that restrict the development of pathogens, including *Staphylococcus aureus*, *Escherichia coli*, *Salmonella typhi*, and *Klebsiella pneumonia* (Kaushik and Chauhan, 2008). *Spirulina* has immunostimulant properties that improve the second humoral response against SRBC antigens of broilers [46,47].

Abdelnour et al. [39] have shown BW, average daily gain, and FCR improvements in rabbits growing under high ambient temperature with the addition of PC at 50, 100, and 150 mg/kg diet. Moreover, the addition of *S. platensis* in the diets improved the BW and BWG but decreased FCR of broilers [36,48,49] and weaned piglets [50]. In contrast, Mirzaie et al. [46] found that adding SP at 0.5, 1, or 2% to diets had no effect on BW, average daily BWG, FI, and FCR of broilers grown under high temperature. Furthermore, prior studies have reported that adding *Spirulina* has a nonsignificant effect on broilers’ performance parameters [51,52,53].

### 4.2. Blood Biochemical Parameters and Lipid Profile

Our findings showed that SPC had no influence on glucose levels, which resembled the findings of Zahir et al. [54], who demonstrated that dietary supplementation with *SP* at 0.5, 1, and 1.5% had no effect on glucose levels in broiler chicken. As the amino acid aspartate found in PC is crucial for protein synthesis and hormone liberation, SPC addition enhanced the serum total proteins, albumin, total globulins, and GH, as well as linear and quadratic increase in thyroxin [55]. Thyroid hormones have an important role in lipid homeostasis by transport and β-oxidation of fatty acids and cholesterol clearance [56]. Thyroid hormones increase the hydroxylase levels that transform cholesterol into bile acids, control cholesterol levels, and inhibit LDL-C apolipoprotein expression, reducing their serum levels [57]. Moreover, thyroid hormones adjust the expression of genes in lipogenesis [58]. Furthermore, the findings showed that SPC treatments lowered cholesterol, LDL-C, and triglycerides levels. Safari et al. [10] demonstrated that C-PC is a potent free radical scavenger that inhibits lipid peroxidation in zero time and sixty days later.

Moreover, previous studies in broilers have demonstrated that *Spirulina* supplementation lowered serum cholesterol levels, triglycerides, total lipids, and LDL compared to the control treatment [46,59,60]. In addition, plasma cholesterol level was reduced with *Spirulina* supplementation [5]. The hypolipidemic effect of SPC may be attributed to decreasing the lipase activity of the pancreas in a dose-dependent manner [7] and enhancing lipid peroxidation [61]. In contrast, Abdelnour et al. [39] found that adding PC to rabbit diets at 50, 100, and 150 mg/kg diet had no effect on triglyceride levels. 

### 4.3. Antioxidant Activity and Inflammatory Markers

The activity of serum antioxidant enzymes (CAT, SOD, and TAC) increased linearly and quadratically. SPC supplementation reduced MDA linearly, which might be attributed to their potent antioxidant activity [62,63,64], resulting from radical-scavenging and metal chelation [65]. These findings are consistent with those of Abdelnour et al. [39], who discovered that adding 50 or 100 mg/kg PC to a rabbit diet increased TAC. Furthermore, Mirzaie et al. [46] and Moustafa et al. [60] showed that birds fed *Spirulina*-containing diets had higher SOD and total antioxidant activities and lower MDA values than those fed basal diets. Furthermore, Park et al., 2018, showed a linear increase in enzyme activities of SOD and GPx by *Spirulina* supplementation at 0.25, 0.5, 0.75, and 1% of broiler diets. 

This study showed that SPC supplementation does not affect IL1β. IL1β is a potent proinflammatory cytokine essential for disease and infection responses [66] and is an integral member of the IL-1 family, secreted by different cell types. The anti-inflammatory properties of PC could be due to its potential to inhibit the synthesis of IL-1, IL-6, and TNF-gamma cytokines and inducible nitric oxide synthase activities (iNOS), enzymes of cyclooxygenase 2 (COX-2) [61], and the strong anti-inflammatory effect of C-phycocyanin [67]. However, adding PC at 50 and 100 mg/kg diet decreased the interleukin-4 and interferon-gamma levels of rabbits [39].

### 4.4. Histological Findings

The duodenum and jejunum play an important role in the digestion and absorption of nutrients in broilers. Well-developed small intestine results in improved nutrient utilization and further improves growth performance [3,68]. This intestinal development is measured using the morphometric measures of the VH and CD, with longer villus and lower CD, resulting in increased mucosal surface area and improved digestive efficacy [68,69]. In addition, the goblet cell count evaluates the small intestine condition [70]. This study showed increased villus height and width, VH: CD ratio, and goblet cells count of small intestine sections by SPC supplementation that suggested its positive effect on gut health, nutrient utilization, and growth. The findings are consistent with previous research on broilers, which stated that SPC supplementation positively affects villi height, CD, and goblet cell numbers of the intestine, improving their nutrients absorption, FCR, and body mass [71,72].

### 4.5. Immunohistochemical Examination

The anti-inflammatory effect of SPC supplementation at different levels showed a decreased immunolabelling index of caspase-3 and TNF-α. TNF-α is an inflammatory cytokine produced by macrophages/monocytes during acute inflammation and is essential in fighting cancer and infection. Caspase-3 is a lysosomal enzyme that destroys specific proteins and is required for the efficient execution of cell apoptosis. The anti-inflammatory properties of PC could be due to its aptitude to downregulate the expression of IL-1β, IL-2, interferon-γ, and TNF-α and increase IL-4 anti-inflammatory cytokines expression [73]. PC is a COX-2 inhibitor with hepatoprotective and anti-inflammatory activities [74]. Its hepatoprotective property is attributed to its ability to inhibit hepatocyte growth factor and TGF-β1 production, obstructing inflammatory infiltration [75]. Martinez et al. [76] demonstrated that PC preparation can limit TNF-α and interleukin-6 (IL-6) and can inhibit the formation of iNOS, COX-2, TNF-α, and neutrophil infiltration into the inflammation site [77].

## 5. Conclusions

The study concludes that SPC supplementation in broiler chicken diets could improve their performance, characterized by increased final BW and BWG and decreased FCR without affecting total FI. Dietary SPC increased the serum levels of total proteins, albumin, total globulins, growth, and thyroxin hormones, without affecting serum glucose and leptin levels. It acts as a hypolipidemic substance that decreases TC, LDL-C, and triglyceride levels, improves the intestinal histology, and enhances the antioxidant activity represented by increased TAC, CAT, and SOD activities. SPC supplementation decreases the MDA level and acts as an anti-inflammatory compound by downregulating the percentage of the immunolabelling index of caspase-3 and TNF-α. Therefore, SPC can be used as an alternative natural growth promoter, antioxidant, and anti-inflammatory feed additive for broilers production.

## Figures and Tables

**Table 1:** Proximate and chemical composition of the experimental diets (%).

Ingredients	Starter Period (4–10 Day)	Grower Period (11–23 Day)	Finisher Period (24–35 Day)
Yellow corn	56	59.3	62
Soybean meal, 48%	33.33	28.1	23.825
Corn gluten, 60%	3.925	5.275	6.07
Soybean oil	2.2	3	4
Calcium carbonate	1.2	1.2	1.1
Calcium dibasic phosphate	1.5	1.4	1.3
Common salt	0.15	0.15	0.15
Premix *	0.3	0.3	0.3
DL-methionine, 98%	0.4	0.3	0.33
Lysine HCl, 78%	0.47	0.45	0.40
Choline	0.07	0.07	0.07
Threonine	0.1	0.1	0.1
Phytase	0.005	0.005	0.005
Na_2_CO_3_	0.25	0.25	0.25
Antimycotoxin	0.1	0.1	0.1
Chemical composition			
ME (Kcal/kg)	3003.9	3100.4	3200.8
Crude protein %	23.01	21.52	20.15
Calcium %	0.941	0.904	0.833
Available phosphorus %	0.481	0.449	0.418
Lysine %	1.465	1.315	1.163
Methionine %	0.721	0.610	0.626
Threonine %	0.823	0.765	0.713

* Premix per kg of diet: Vitamin A, 1 500 IU; Vitamin D3, 200 IU; Vitamin E, 10 mg; Vitamin K3, 0.5 mg; thiamine, 1.8 mg; riboflavin, 3.6 mg; pantothenic acid, 10 mg; folic acid, 0.55 mg; pyridoxine, 3.5 mg; niacin, 35 mg; cobalamin, 0.01 mg; biotin, 0.15 mg; Fe, 80 mg; Cu, 8 mg; Mn, 60 mg; Zn, 40 mg; I, 0.35 mg; Se, 0.15 mg.

**Table 2:** Effects of dietary *Spirulina platensis* phycocyanin on the growth performance of broiler chicken.

Trait Studied	SPC0	SPC0.25	SPC0.5	SPC0.75	SPC1	Regression ^#^	
						Linear	Quadratic
Initial BW (g)	88.96 ± 0.95	89.17 ± 1.30	90.00 ± 0.63	89.59 ± 0.96	89.59 ± 0.36	0.333	0.41
Starter Period (4–10 Days)							
BW (g)	320.60 ± 4.93 ^bc^	315.61 ± 7.64 ^c^	333.25 ± 8.39 ^ab^	331.23 ± 9.78 ^ab^	337.81 ± 7.59 ^a^	<0.01	0.84
BWG (g)	231.64 ± 5.78 ^bc^	226.44 ± 6.41 ^c^	243.26 ± 8.94 ^ab^	241.65 ± 9.01 ^ab^	248.23 ± 7.66 ^a^	<0.01	0.76
FI (g)	240.84 ± 6.26	236.88 ± 16.16	241.25 ± 9.44	247.29 ± 12.35	238.13 ± 15.27	0.83	0.75
FCR	1.04 ± 0.01	1.05 ± 0.07	0.99 ± 0.02	1.02 ± 0.06	0.96 ± 0.07	0.09	0.62
Grower Period (11–23 Days)							
BW (g)	1140.97 ± 0.87 ^bc^	1100.90 ± 15.89 ^c^	1211.72 ± 25.68 ^ab^	1243.33 ± 67.66 ^a^	1205.17 ± 47.86 ^ab^	<0.01	0.40
BWG (g)	820.37 ± 5.46 ^bc^	785.30 ± 23.30 ^c^	878.47 ± 28.36 ^ab^	912.10 ± 60.87 ^a^	867.36 ± 40.88 ^ab^	0.01	0.34
FI (g)	1070.09 ± 16.56 ^ab^	1170.36 ± 52.13 ^a^	1042.92 ± 26.25 ^b^	1091.25 ± 63.36 ^ab^	1077.08 ± 75.44 ^ab^	0.51	0.65
FCR	1.30 ± 0.02 ^b^	1.49 ± 0.05 ^a^	1.19 ± 0.07 ^c^	1.20 ± 0.06 ^c^	1.24 ± 0.05 ^bc^	<0.01	0.86
Finisher Period (24–35 Days)							
BW (g)	2126.22 ± 32.50 ^b^	2363.89 ± 38.08 ^a^	2487.50 ± 78.70 ^a^	2499.17 ± 80.55 ^a^	2455.83 ± 82.17 ^a^	<0.01	<0.01
BWG (g)	985.25 ± 31.82 ^b^	1262.99 ± 127.54 ^a^	1275.78 ± 53.23 ^a^	1255.83 ± 80.86 ^a^	1250.67 ± 54.17 ^a^	<0.01	<0.01
FI (g)	1709.82 ± 105.53	1710.12 ± 127.23	1736.51 ± 233.40	1752.08 ± 165.63	1748.61 ± 185.14	0.71	0.96
FCR	1.73 ± 0.08	1.37 ± 0.23	1.36 ± 0.13	1.40 ± 0.21	1.40 ± 0.14	0.06	0.05
Overall Performance (1–35 Days)							
Final BW, g	2126.22 ± 32.50 ^b^	2363.89 ± 138.08 ^a^	2487.50 ± 78.70 ^a^	2499.17 ± 80.55 ^a^	2455.83 ± 82.17 ^a^	<0.01	0.01
Total BWG, g	2037.27 ± 32.11 ^b^	2274.72 ± 138.99 ^a^	2397.50 ± 78.26 ^a^	2409.58 ± 80.04 ^a^	2366.25 ± 81.89 ^a^	<0.01	0.01
Total FI, g	3020.75 ± 85.47	3117.35 ± 142.64	3020.68 ± 216.28	3090.63 ± 238.36	3063.82 ± 257.01	0.87	0.86
FCR	1.48 ± 0.03 ^a^	1.38 ± 0.14 ^ab^	1.26 ± 0.05 ^b^	1.28 ± 0.11 ^b^	1.30 ± 0.08 ^b^	0.02	0.09

^#^ The regressions were considered significant at *p* ≤ 0.05. ^a,b,c^ Means within the same row carrying different superscripts were significantly different at *p* ≤ 0.05. SPC0: control diet (basal diet with no additives); SPC0.25: basal diets supplemented with 0.25 g SPC kg^–1^ diet; SPC0.5: basal diets supplemented with 0.5 g SPC kg^–1^ diet; SPC0.75: basal diets supplemented with 0.75 g SPC kg^–^^1^ diet; SPC1: basal diets supplemented with 1 g SPC kg^–^^1^ diet. BW: body weight, BWG: body weight gain, FI: feed intake, and FCR: feed conversion ratio.

**Table 3:** Effect of dietary *Spirulina platensis* phycocyanin addition on serum biochemical parameters.

	SPC0	SPC0.25	SPC0.5	SPC0.75	SPC1	Regression ^#^	
						Linear	Quadratic
Glucose (mg/dL)	335.00 ± 2.65	337.33 ± 7.57	339.33 ± 6.43	338.67 ± 6.43	340.33 ± 6.11	0.30	0.77
Total proteins (g/dL)	3.21 ± 0.25 ^b^	4.32 ± 0.89 ^a^	4.69 ± 0.36 ^a^	4.88 ± 0.53 ^a^	5.05 ± 0.27 ^a^	<0.01	0.10
Albumin (g/dL)	1.21 ± 0.03 ^c^	1.42 ± 0.13 ^bc^	1.54 ± 0.17 ^b^	1.87 ± 0.31 ^a^	2.02 ± 0.04 ^a^	<0.01	0.79
Total globulins (g/dL)	2.00 ± 0.24 ^b^	2.90 ± 0.80 ^a^	3.15 ± 0.28 ^a^	3.02 ± 0.25 ^a^	3.03 ± 0.24 ^a^	0.02	0.05
GH (ng/mL)	2.90 ± 0.30 ^c^	4.20 ± 0.30 ^b^	4.90 ± 0.44 ^ab^	5.30 ± 0.46 ^a^	5.60 ± 0.72 ^a^	<0.01	0.05
Leptin (ng/mL)	2.18 ± 0.06	2.19 ± 0.02	1.65 ± 0.51	2.02 ± 0.32	2.06 ± 0.52	0.54	0.24
T3 (ng/mL)	3.44 ± 0.21 ^c^	4.42 ± 0.10 ^b^	4.29 ± 0.11 ^b^	4.41 ± 0.10 ^b^	4.85 ± 0.22 ^a^	<0.01	0.03
T4 (ng/mL)	18.55 ± 1.47 ^c^	23.06 ± 0.57 ^b^	24.62 ± 0.88 ^ab^	25.12 ± 1.02 ^a^	24.41 ± 0.25 ^ab^	<0.01	<0.01

^#^ The regressions were considered significant at *p* ≤ 0.05. ^a,b,c^ Mean values in the same row with different superscripts differ significantly (*p* < 0.05). SPC0: control diet (basal diet with no additives); SPC0.25: basal diets supplemented with 0.25 g SPC kg^–1^ diet; SPC0.5: basal diets supplemented with 0.5 g SPC kg^–1^ diet; SPC0.75: basal diets supplemented with 0.75 g SPC kg^–1^ diet; SPC1: basal diets supplemented with 1 g SPC kg^–1^ diet. GH: growth hormone, T3: triiodothyronine, and T4: thyroxine hormone.

**Table 4:** Effect of dietary *Spirulina platensis* phycocyanin addition on serum lipid profile.

	SPC0	SPC0.25	SPC0.5	SPC0.75	SPC1	Regression ^#^	
						Linear	Quadratic
TC (mmol/L)	3.54 ± 0.06 ^a^	3.35 ± 0.01 ^c^	3.46 ± 0.05 ^ab^	3.48 ± 0.03 ^a^	3.39 ± 0.02 ^bc^	0.04	0.23
HDL-C (mmol/L)	1.98 ± 0.03	2.07 ± 0.12	2.07 ± 0.13	2.08 ± 0.08	2.13 ± 0.09	0.11	0.79
LDL-C (mmol/L)	1.32 ± 0.03 ^a^	1.05 ± 0.13 ^b^	1.17 ± 0.15 ^ab^	1.16 ± 0.12 ^ab^	1.03 ± 0.09 ^b^	0.04	0.50
VLDL-C (mmol/L)	0.23 ± 0.02	0.23 ± 0.01	0.23 ± 0.02	0.24 ± 0.01	0.23 ± 0.02	0.91	0.93
TG (mmol/L)	1.27 ± 0.04 ^a^	1.17 ± 0.04 ^b^	1.19 ± 0.04 ^ab^	1.15 ± 0.03 ^b^	1.17 ± 0.07 ^b^	0.02	0.09

^#^ The regressions were considered significant at *p* ≤ 0.05. ^a,b,c^ Mean values in the same row with different superscripts differ significantly (*p* < 0.05). SPC0: control diet (basal diet with no additives); SPC0.25: basal diets supplemented with 0.25 g SPC kg^–1^ diet; SPC0.5: basal diets supplemented with 0.5 g SPC kg^–1^ diet; SPC0.75: basal diets supplemented with 0.75 g SPC kg^–1^ diet; SPC1: basal diets supplemented with 1 g SPC kg^–1^ diet. TC: total cholesterol, TG: triglycerides, HDL-C: high-density lipoprotein cholesterol, LDL-C: low-density lipoprotein cholesterol, and VLD-CL: very low-density lipoprotein cholesterol.

**Table 5:** Effect of dietary *Spirulina platensis* phycocyanin (SPC) addition on serum antioxidant and inflammatory indices.

	SPC0	SPC0.25	SPC0.5	SPC0.75	SPC1	Regression ^#^	
						Linear	Quadratic
TAC (U/mL)	9.69 ± 0.63 ^c^	11.59 ± 1.51 ^b^	13.11 ± 0.81 ^ab^	13.12 ± 0.46 ^ab^	13.69 ± 0.41 ^a^	<0.01	0.04
CAT (U/mL)	2.57 ± 0.45 ^b^	5.03 ± 1.92 ^a^	6.50 ± 0.57 ^a^	6.43 ± 0.55 ^a^	6.57 ± 0.60 ^a^	<0.01	0.02
SOD (U/mL)	135.96 ± 3.34 ^b^	152.21 ± 8.28 ^a^	156.32 ± 1.97 ^a^	162.44 ± 3.39 ^a^	153.49 ± 9.03 ^a^	0.01	0.02
MDA nmol/mL	4.59 ± 0.39 ^a^	4.60 ± 1.18 ^a^	3.63 ± 0.75 ^b^	3.17 ± 0.90 ^b^	3.43 ± 0.71 ^b^	0.03	0.59
Serum IL1β (μg/mL)	155 ± 11.79	157 ± 12.12	142 ± 6.08	157 ± 15.13	165 ± 7.94	0.35	0.11

^#^ The regressions were considered significant at *p* ≤ 0.05. ^a,b,c^ Mean values in the same column with different superscripts differ significantly (*p* < 0.05). TAC: total antioxidant capacity, CAT: catalase, SOD: superoxide dismutase, MDA: malondialdehyde, and IL1β: interleukin 1β. SPC0: control diet (basal diet with no additives); SPC0.25: basal diets supplemented with 0.25 g SPC kg^–1^ diet; SPC0.5: basal diets supplemented with 0.5 g SPC kg^–1^ diet; SPC0.75: basal diets supplemented with 0.75 g SPC kg^–1^ diet; SPC1: basal diets supplemented with 1 g SPC kg^–1^ diet.

**Table 6:** Effect of dietary *Spirulina platensis* phycocyanin addition on intestinal histology.

	SPC0	SPC0.25	SPC0.5	SPC0.75	SPC1	Regression ^#^	
						Linear	Quadratic
Duodenum							
VH µm	626.55 ± 36.97 ^d^	706.20 ± 31.64 ^c^	921.23 ± 40.00 ^b^	1042.44 ± 20.14 ^a^	922.34 ± 29.14 ^b^	<0.01	<0.01
VW µm	130.36 ± 1.64	82.46 ± 35.09	115.57 ± 41.43	118.39 ± 9.57	65.51 ± 15.68	0.07	0.64
CD µm	115.26 ± 13.38 ^d^	119.01 ± 19.78 ^cd^	153.78 ± 24.18 ^b^	232.42 ± 13.51 ^a^	148.40 ± 7.78 ^b^	<0.01	<0.01
VH:CD	4.66 ± 0.44 ^c^	5.85 ± 0.32 ^ab^	5.81 ± 0.39 ^ab^	5.49 ± 0.18 ^b^	6.29 ± 0.21 ^a^	<0.01	0.02
GCC	100.70 ± 11.84 ^c^	125.90 ± 11.64 ^b^	139.23 ± 6.60 ^ab^	155.59 ± 5.16 ^a^	131.39 ± 10.24 ^b^	<0.01	<0.01
Jejunum							
VH µm	854.95 ± 77.77 ^d^	995.13 ± 66.11 ^c^	1213.0 ± 38.47 ^b^	1373.18 ± 104.11 ^a^	1038.93 ± 56.37 ^c^	<0.01	<0.01
VW µm	83.30 ± 14.85	98.86 ± 8.94	95.14 ± 5.05	93.77 ± 8.11	90.82 ± 17.21	0.36	0.17
CD µm	163.78 ± 6.42 ^d^	210.36 ± 24.55 ^c^	254.38 ± 16.90 ^b^	321.66 ± 17.43 ^a^	195.31 ± 13.12 ^c^	<0.01	<0.01
VH:CD	1.28 ± 0.09 ^c^	5.18 ± 0.93 ^a^	4.78 ± 0.19 ^ab^	4.27 ± 0.11 ^b^	5.42 ± 0.18 ^a^	<0.01	<0.01
GCC	170.45 ± 17.64 ^c^	210.36 ± 24.55 ^b^	242.98 ± 22.74 ^b^	298.98 ± 13.12 ^a^	210.45 ± 11.48 ^b^	<0.01	<0.01
Ileum							
VH µm	254.76 ± 44.46 ^c^	382.31 ± 14.48 ^b^	392.03 ± 10.37 ^b^	506.08 ± 41.73 ^a^	349.00 ± 28.15 ^b^	<0.01	<0.01
VW µm	72.72 ± 7.33 ^c^	107.17 ± 5.56 ^ab^	74.97 ± 11.78 ^c^	91.41 ± 9.98 ^bc^	124.69 ± 16.28 ^a^	<0.01	0.07
CD µm	86.15 ± 8.35 ^c^	92.34 ± 4.22 ^bc^	100.49 ± 2.51 ^ab^	110.11 ± 10.54 ^a^	87.73 ± 2.56 ^c^	0.09	<0.01
VH:CD	626.55 ± 36.97 ^d^	3.93 ± 0.22 ^b^	3.90 ± 0.18 ^b^	4.60 ± 0.17 ^a^	3.98 ± 0.24 ^b^	<0.01	<0.01
GCC	130.36 ± 1.64	71.56 ± 6.23 ^b^	80.55 ± 2.57 ^ab^	89.01 ± 4.02 ^a^	74.01 ± 5.14 ^b^	<0.01	<0.01

^#^ The regressions were considered significant at *p* ≤ 0.05. ^a,b,c,d^ Means within the same row carrying different superscripts were significantly different (*p* ≤ 0.05). SPC0: control diet (basal diets with no additives); SPC0.250: basal diets supplemented with 0.250 g SPC kg^–^^1^ diet; SPC0.5: basal diets supplemented with 0.5 g SPC kg^–^^1^ diet; SPC0.75: basal diets supplemented with 0.75 g SPC kg^–^^1^ diet; SPC1: basal diets supplemented with 1 g SPC kg^–^^1^ diet. VH: villus height, VW: villus width, CD: crypt depth, and GCC: goblet cell count.

## Data Availability

Data contained in the article.
